# Evaluation of GABA Production by Alginate-Microencapsulated Fresh and Freeze-Dried Bacteria Enriched with Monosodium Glutamate during Storage in Chocolate Milk

**DOI:** 10.3390/microorganisms11112648

**Published:** 2023-10-28

**Authors:** Hebat Allah Ibrahim Youssef, Paola Vitaglione, Rosalia Ferracane, Jumana Abuqwider, Gianluigi Mauriello

**Affiliations:** 1 Microbiology Department, Faculty of Science, Ain Shams University, El-Khalyfa El-Mamoun St. Abbasya, Cairo 11566, Egypt; hoba83@sci.asu.edu.eg; 2 Department of Agricultural Sciences, University of Naples Federico II, Via Università 100, 80055 Portici, Italy; paola.vitaglione@unina.it (P.V.); rosalia.ferracane@unina.it (R.F.); jqwider@gmail.com (J.A.)

**Keywords:** gamma-amino butyric acid, gut–brain axis, microencapsulation, psychobiotic, vibrating technology

## Abstract

Two strains of γ-aminobutyric acid (GABA) producing bacteria, *L. brevis* Y1 and *L. plantarum* LM2, were microencapsulated in sodium alginate with two concentrations (1% and 2%) of monosodium glutamate (MSG) by using vibrating technology. The mix of both species was microencapsulated both in fresh and freeze-dried form. After 0, 1, 2, and 4 weeks of storage at 4 °C in quarter strength Ringer’s solution, the microcapsules were subjected to cell viable counting and sub-cultured in MRS at 37° for 24 h. The MRS cultures were analyzed for the GABA content. The amount of GABA produced per CFU of MRS inoculum was then calculated. Only the 4-week-old microcapsules were used to inoculate a chocolate milk drink with the aim of obtaining a functionalized drink containing viable probiotic cells and GABA after a 1-week incubation at 4 °C. Therefore, the GABA production in chocolate milk per CFU of the probiotic culture after the incubation time was calculated. Results of the GABA analysis by liquid chromatography mass spectrometry of the MRS sub-cultures showed no significant difference (*p* > 0.05) in GABA yield between 1% and 2% MSG for the microcapsules containing fresh cells. On the contrary, a significant difference (*p* < 0.05) in productivity along the storage was registered. Microcapsules containing freeze-dried cells showed significant differences (*p* < 0.05) in GABA yield between 1% and 2% MSG only after 2 and 4 weeks of storage. A significant difference (*p* < 0.05) in GABA yield between the storage time was found only for the trials with 2% MSG for freeze-dried cells. The synthesis of GABA in chocolate milk significantly decreased (*p* < 0.05) only for fresh cells when comparing 2% with 1% MSG. In conclusion, a 1-month storage of microcapsules containing both culture forms, fresh and freeze-dried, did not affect GABA production.

## 1. Introduction

The γ-aminobutyric acid (GABA) is a potential metabolic bioactive ingredient with increasing evidence of involvement in the gut–brain axis and systemic metabolic health [1]. GABA works as an inhibitory neurotransmitter in the brain; it can exert its effect by regulating mood and relaxation, enhancing memory and sleep disorders, as well as having a beneficial effect on depression, asthma, and many other diseases. It also plays a vital role in cardiovascular diseases, diabetes, cancer, and drug addiction [1]. GABA is used as an ingredient in some foods to confer antihypertensive and antidepressant activities, so it has a crucial role in neurological disorders. Moreover, GABA-producing bacteria are known as “psychobiotics” [2]. Psychobiotics are defined as probiotics that confer mental health benefits to the host when consumed in a particular quantity through the interaction with commensal gut bacteria [3]. Adikari et al. have investigated GABA’s effect in alleviating anxiety, sleeplessness, and controlling energy metabolism [2]. On the other hand, it is synthesized as an anti-stress protective mechanism in plants and some microorganisms [4]. As well established, GABA is formed from the decarboxylation of L-glutamate by glutamate decarboxylase [5]. Numerous microorganisms can synthesize many amino acids, which makes it possible to produce GABA from endogenous L-glutamate [6]. The majority of LAB, however, are unable to produce enough L-glutamate for GABA synthesis. Due to the ease with which monosodium glutamate (MSG) can be hydrolyzed into L-glutamate, its addition to culture media is essential [7]. Different lactic acid bacteria produce GABA, particularly strains of *Lactobacillus brevis* [8] and *Lactobacillus plantarum* [9]. Although GABA-producing lactobacilli are recognized as positive for human health, they are susceptible to perishing under storage conditions, which would result in decreased viability and GABA production [10]. Accordingly, during the production of probiotic food, the viability of the cells within the food and their bioavailability upon consumption by the host must be considered [11]. Freeze-drying is a common method to ensure the storage stability of bacterial cells, in general, and of probiotics as well. However, processing and storage decrease the freeze-dried probiotic bacteria vitality. The main problem is finding protective agents that improve cellular life during their storage and application in food [12]. Microencapsulation is a promising technique for the protection of probiotic bacteria [13] in several industries, and especially in the food sector [14]. The microencapsulation process can protect microbial cells from adverse environmental conditions through entrapment within a matrix of biopolymeric material [15]. The microencapsulation material should be natural; permeable to nutrients and metabolites, to provide optimal conditions for the functionality of the entrapped bacteria; and biodegradable, to ensure the release of the cells in the host colon [16]. Techniques commonly applied for probiotic microencapsulation are emulsion, extrusion, spray drying, freeze drying and adhesion to starch. A recent comprehensive review on the microencapsulation of probiotics, including the description of microencapsulation techniques, was published by Rajam and Subramanian in 2022 [17].

One microencapsulation method for probiotic bacteria is the use of vibrating technology. This technique can yield monodispersed droplets of different sizes. The size can be determined by the nozzle diameter and the frequency of the sinusoidal force applied. The droplets can be collected in a hardening solution such as calcium chloride. This technology has many benefits, including a modest size dispersion (5–10%), the ability to operate in sterile settings, and a high flow rate (0.1–2 L/h) [18].

Microcapsules can also be carriers of compounds which have an additional beneficial health effect, such as prebiotics, which allows the design of symbiotic microcapsules [19]. Within this framework, some authors have entrapped both probiotics and GABA in microbeads to confer super health activity to this product and to improve the gut–brain axis [20,21,22,23]. Some authors have also entrapped GABA-producer strains in microbeads [24,25].

When it comes to using microencapsulation for adding probiotics to food, the interaction with the matrix also matters. For instance, *Lactobacillus brevis* is more stable in milk chocolate than in probiotic powders [26]. The complex mixture of proteins, polysaccharides, and lipids that makes up cocoa powder is one of the key components of chocolate production [27]. All over the world, people of all ages and social groups eat chocolate. Therefore, it is not surprising that chocolate milk could be used as a probiotic carrier [28]. Notably, flavonoids and polyphenols, two physiologically active chemicals with substantial antioxidant potential, are particularly present in chocolate milk. As far as we are aware, this is the first published investigation aimed at microencapsulating GABA-producing bacteria and the GABA-precursor monosodium glutamate. Therefore, this is the main novelty of this paper, which is aimed at preserving the survival of the probiotic strains and improving the biosynthesis of GABA. Moreover, the inclusion in chocolate milk of microcapsules containing GABA-producer strains and the GABA precusor is the final goal of this work.

## 2. Materials and Methods

### 2.1. Chemicals, Media, and Microorganisms

All chemicals and media used in this research were from (Oxoid, Madrid, Spain); which include De Man Rogosa and Sharpe agar (MRS) (CM0359), Ringer’s solution (BR0052), monosodium glutamate (MSG) (LP0124), calcium chloride (CaCl_2_) (CM0519), and phosphate buffer saline (PBS) (BR0014). Sodium citrate (W302600) and sodium alginate (NaAlg; A0682) were from Sigma-Aldrich, St. Louis, MO, USA.

Two lactic acid bacteria strains, *Lactiplantibacillus plantarum* LM2 and *Levilactobacillus brevis* Y1, were isolated from the commercial supplement Gabaflor (Proge Farm, Largo Donegani 4/A, Novara, Italy). The identity of the microorganisms at the species level was confirmed by 16S rRNA sequencing of two different cultures, according to the morphology of colonies grown on MRS agar. 

### 2.2. Freeze Drying

The two strains of lactic acid bacteria were sub-cultured twice and then grown overnight at 37 °C in 200 mL MRS broth. The broth was centrifuged at 4500× *g* for 15 min and washed twice with PBS. The pellets of each bacterium were mixed separately with sterile skimmed milk solution (11%) at a ratio of 1:5 (1 mL milk for 5 g of concentrated cells) and kept in the freezer at −18 °C overnight. The samples were then freeze-dried at −80 °C for 24 h using an Edwards Madulyo freeze-dryer (Bristol, UK).

### 2.3. Microencapsulation by Vibrating Technology

Microencapsulation was performed on both fresh and lyophilized cells. The process was conducted according to De Prisco et al. [18] by using the Encapsulator B-395 Pro supplemented with a 120 µm nozzle and a syringe pump. Overnight cultures of *L. brevis* Y1 and *L. plantarum* LM2 were harvested by centrifugation at 4500× *g* for 15 min. After that, the pellets of the fresh cells were washed twice in an equal volume of a sterile quarter strength Ringer’s solution (Ringer). The cells were harvested by centrifugation and finally suspended in an equal volume of a 12 g/L sterile sodium alginate solution supplemented with 1 and 2% of MSG. Fifty ml of the alginate cell suspension (25 mL of *L. brevis* Y1 and 25 mL *L. plantarum* LM2) were well mixed and loaded into the syringe and placed in the encapsulator. A flow rate of 2.50 mL/min was used in the process, with a vibration frequency of 1300 Hz and electrode voltage of 1800 V. A sterile plate containing 200 mL solution of CaCl_2_ was used to collect the suspension of alginate with bacterial cells at a ratio of 4:1. The suspension was stirred by a magnet in CaCl_2_ continuously for 20 min until cross-linked monodispersed microcapsules were formed. To restore the initial cell concentration, the suspension was kept at room temperature for about 30 min to allow settlement of the microcapsules, and a volume of 150 mL of the suspension was discarded. Finally, it was returned to its original volume by adding Ringer’s solution and then kept in the refrigerator at 4 °C. Freeze-dried powders of both cultures were mixed at a ratio of 1:1 and resuspended in 50 mL of sodium alginate solution with either 1 or 2% MSG and microencapsulated as previously described.

### 2.4. Screening of GABA Production after Microcapsule Storage

The microcapsules were stored for one month at 4 °C in Ringer, and their cell viability and GABA-production ability were assessed every week. After the end of each week, the cell load was assessed, and the microcapsules were inoculated in MRS broth and incubated for 48 h at 37 °C to test the GABA production. The cell load of microcapsules was assessed by suspending 1 mL of microcapsules into 9 mL of 0.06 M sodium citrate for 5 min to destroy the calcium alginate network and promote the release of cells. Afterward, decimal serial dilution in Ringer, spreading onto MRS agar, and plate incubation at 37 °C for 48 h was performed [18]. An aliquot of MRS inoculated with the microcapsules was collected after the incubation period. This aliquot was centrifuged at 4500× *g* for 15 min, and the supernatant was harvested and transferred to a sterile and clean tube to be analyzed for GABA content by liquid chromatography coupled with tandem mass spectrometry (LC/MS/MS). 

### 2.5. GABA Analysis by LC/MS/MS

LC/MS/MS analysis was carried out using a briefly modified version of the method reported by Wu and co-workers [29]. The system consisted of an HPLC apparatus equipped with two Series 200 micropumps (Perkin Elmer, Shelton, CT, USA), a Kinetex 2.6 µ HILIC 100 Å column (100 mm × 2.1 mm) (Phenomenex, Torrance, CA, USA) thermostatically controlled at 30 °C, and an API 3000 triple quadrupole mass spectrometer (Applied Biosystems, Mississauga, ON, Canada) equipped with a TurboIon Spray source. The mobile phase A consisted of H_2_O/CH_3_CN/ammonium acetate 50 mM (50:40:10); mobile phase B consisted of CH_3_CN/ammonium acetate (90:10). The gradient program was as follows: 100% B (2 min), 100-0% B (2–6 min), 0% B (6–10 min), 0-100% B (10–13 min). The flow rate was 0.2 mL/min, and the injection volume was 5 μL. Drying gas (air) was heated to 400 °C, and the capillary voltage (IS) and the declustering potential (DP) were set to 5500 V and 30 V, respectively. Analysis was performed in the positive ion mode in Multiple Reaction Monitoring (MRM) using the following precursor/product ion combination: *m*/*z* 104→87. The detection limit (LOD) for GABA was 5 ppb, and the quantification limit (LOQ) was 10 ppb. The calibration curve was constructed within the linearity range of 10–500 ng/mL. Samples were prepared according to the method described by Luang-In and co-workers [30], with slight modifications. Culture broths were centrifuged at 14,800× *g* at 4 °C for 10 min, filtered with 0.22 µm regenerated cellulose filters, opportunely diluted with H_2_O/CH_3_CN 0.1% acetic acid solution, and used directly for LC/MS/MS analysis.

### 2.6. Application of Microcapsules in Chocolate Milk

Ten mL of a 4-week-old Ringer microcapsule suspension, containing fresh or freeze-dried cells and mixed with 1% or 2% MSG, was inoculated into 100 mL chocolate milk and stored at 4 °C for 1 week. After this period, the GABA production and viability of the cells were assessed.

### 2.7. Statistical Analysis

All analyses were performed in triplicate, and the vitality of the cells after microencapsulation was evaluated as an average. To determine the significant differences between averages, a one-way ANOVA test (IBM SPPS version 21) and a *t*-test analysis (Microsoft Excel, 2018 version) were used.

## 3. Results

### 3.1. Survival of Probiotic Cells along One Month of Storage at 4 °C in Ringer

The storage in Ringer of the alginate microcapsules, containing the GABA-producing bacteria and supplemented with 1 or 2% MSG, led to a slight reduction in the viable count of cells over 1 month, as shown in Table 1 and Table 2.

### 3.2. Screening of GABA Production after Microcapsule Storage in Ringer

**Figure 1:**
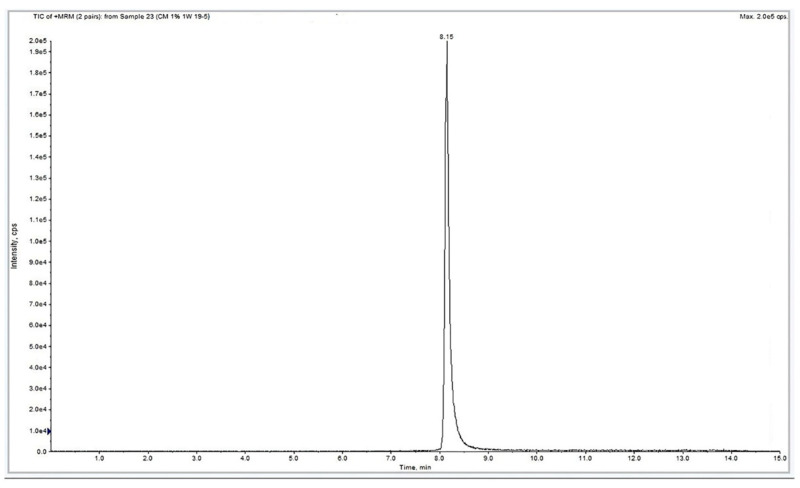
Typical LC/MS/MS chromatogram of analyzed samples.

A typical HPLC chromatogram of the MRS supernatants collected from microcapsule cultures showing the GABA peak is reported in Figure 1. The amount of GABA found in each analyzed sample is reported in Table 3 and Table 4. The GABA values for fresh cells range between 155.5 ± 0.7 µg/mL (2 weeks at 1% MSG) and 273.5 ± 0.7 µg/mL (4 weeks at 1% MSG). The GABA values for freeze-dried cells range between 224 ± 2.8 µg/mL (0 weeks at 2% MSG) and 311.5 ± 2.1 µg/mL (2 weeks at 1% MSG).

**Figure 2:**
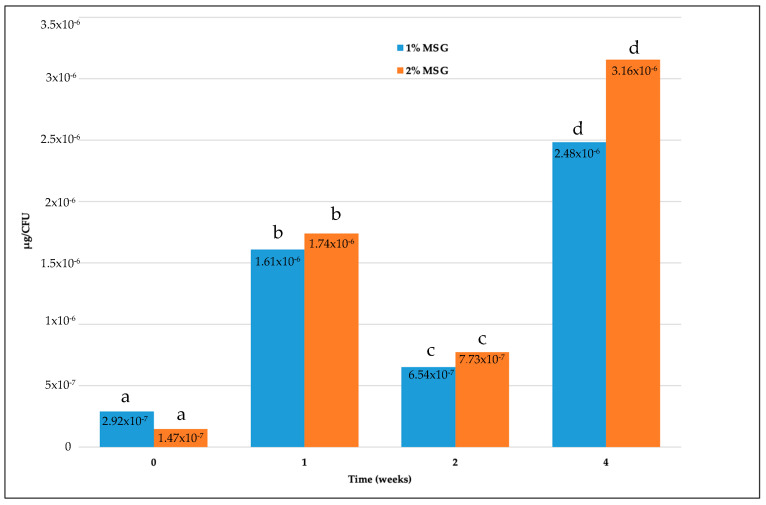
GABA production (µg/CFU) from microencapsulated fresh cells with 1% and 2% MSG after different times of storage at 4 °C in Ringer and additional growth in MRS broth at 37 °C for 48 h. Values are the average of three replicates. Different letters indicate a significant difference at *p* < 0.05, as determined by the *t*-test (values at the same time) and ANOVA and post hoc Tukey tests (values over the time period).

**Figure 3:**
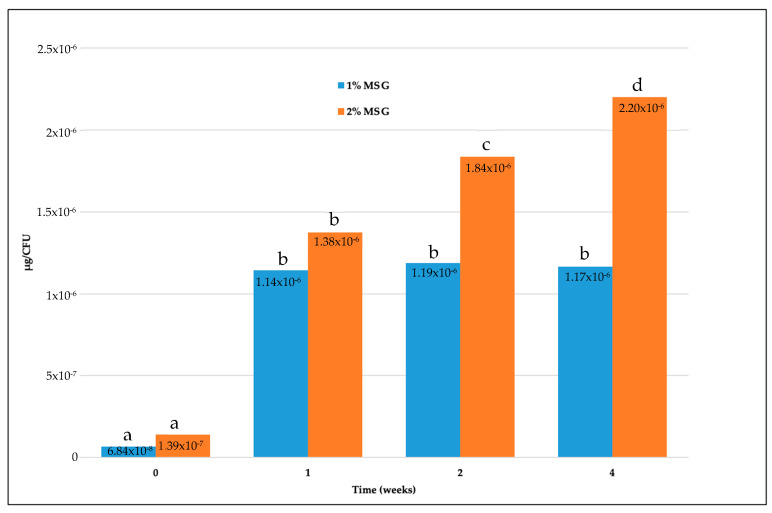
GABA production (µg/CFU) from microencapsulated freeze-dried cells with 1% and 2% MSG, after different times of storage at 4 °C in Ringer and additional growth in MRS broth at 37 °C for 48 h. Values are the average of three replicates. Different letters indicate significant differences at *p* < 0.05, as determined by *t*-test (values at the same time) and ANOVA and post hoc Tukey tests (values over the time period).

In an attempt to evaluate the ability of microencapsulated cells to produce GABA, the cell biomass of both strains at the end of each week and the amount of GABA produced from that biomass after growth in MRS were considered. Therefore, the quantity of GABA per CFU was calculated. Data are visualized in Figure 2 and Figure 3 for the fresh and freeze-dried cells, respectively. Our results showed that the GABA production per CFU significantly (*p* < 0.01) increased according to the time of storage, while no significant difference (*p* > 0.05) was registered when comparing the microcapsules with 1 and 2% MSG.

As shown in Figure 1, the amount of GABA per UFC produced by microencapsulated fresh cells with 1% MSG increased about 10-fold when comparing cells immediately after the microencapsulation (2.92 × 10^−7^ μg/CFU) and 4-week-old cells (2.48 × 10^−6^ μg/CFU). A slight reduction in GABA production was observed by comparing the 1- and 2-week-old cells, with both 1 and 2% MSG.

GABA production per CFU by the microencapsulated freeze-dried cells was also examined (Figure 3). Our findings showed that the amount of GABA per CFU produced in MRS increased with the age of cells. In particular, when 1% of MSG was included in the microcapsules, the amount significantly (*p* < 0.01) increased in the 1-week-old cells compared with the cells immediately after the microencapsulation. However, the amount did not change significantly (*p* > 0.05) until the fourth week of storage. On the other hand, microcapsules with 2% of MSG promoted a continuous increase in the amount of GABA per CFU produced according to the age of the cells. Like microencapsulated fresh cells, the lowest GABA production was at time zero (6.84 × 10^−8^ μg/CFU and 1.39 × 10^−7^ μg/CFU with 1% and 2% MSG, respectively) and increased in the following weeks, reaching higher quantities with 2% than with 1% MSG. Using 2% MSG, the quantity was increased to 1.24 × 10^−6^ μg/CFU in the first week and reached 1.84 × 10^−6^ μg/CFU in the second week. The highest amount was 2.20 × 10^−6^ μg/CFU at the end of the storage time. Samples with 1% MSG also showed a high increase in the production of GABA within the first week of storage as this was enhanced to 1.08 × 10^−6^ μg/CFU. However, in the second and fourth weeks the production per CFU was almost stable, with 1.19 × 10^−6^ μg/CFU in the second week and 1.17 × 10^−6^ μg/CFU in the last week.

### 3.3. Application of Microcapsules in Chocolate Milk Drink

After the addition of microencapsulated fresh and freeze-dried cells to the chocolate milk, GABA production was determined after 1 week of storage at 4 °C (Table 4).

Microcapsules in chocolate milk yielded GABA ranging from 84.8 ± 3.9 µg/mL (by fresh cells microencapsulated with 1% MSG) to 204.5 ± 5.7 µg/mL (by microencapsulated freeze-dried cells, independently of MSG content). Hence, our results showed that microencapsulated freeze-dried cells in chocolate milk have the highest biotransformation potential of MSG to GABA.

The viable count of both strains after 1 week of storage of the chocolate milk at 4 °C was carried out, and the results are reported in Table 5. Moreover, the pH of the chocolate milk was monitored at the end of the storage period, showing a slight reduction from 6.8 to 6.6.

**Figure 4:**
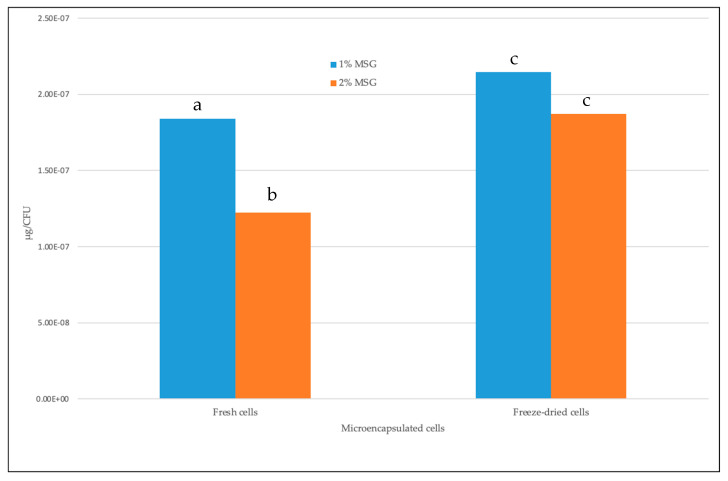
Production of GABA per CFU by microencapsulated fresh and freeze-dried cells in chocolate milk. Values are the average of three replicates. Different letters indicate significant differences at *p* < 0.01, as determined by the *t*-test.

Considering the cell counts after one week of storage in chocolate milk, GABA production from freeze-dried cells using 1% or 2% MSG was higher than that from fresh cells. Also, starting with microcapsules containing the same initial count throughout the study, the application of microencapsulated fresh and freeze-dried cells in chocolate milk showed stable viable counts. The highest amount produced was from using 1% MSG by freeze-dried cells, which was 2.15 × 10^−7^ μg/CFU (*p* = 0.0059). By incorporating 2% MSG, the production was slightly reduced to 2.76 × 10^−8^ μg/CFU (*p* = 0.001). Fresh cells showed lower GABA production which was 1.84 × 10^−7^ μg/CFU in 1% MSG and 1.22 × 10^−7^ μg/CFU in 2% MSG respectively (Figure 4). There is a significant difference (*p* < 0.01) in the amount of GABA produced from 1 and 2% MSG by microencapsulated fresh cells, while no difference (*p* > 0.05) was registered between freeze-dried cells.

## 4. Discussion

GABA is the product of glutamic acid decarboxylation in lactic acid bacterial cells by glutamic acid decarboxylase (GAD). GABA production begins in the bacterial growth phase and increases near the stationary phase due to the increased activity of GAD. This is an intracellular enzyme produced in response to an acidic environment [31]. MSG is an ideal substrate for GABA production. Decarboxylation of glutamate to GABA leads to the release of carbon dioxide that coincides with the consumption of hydrogen (H^+^); as a result, an increase in the pH of the medium happens [32]. Yogeswara et al. [33] isolated thirty strains of GABA producing lactic acid bacteria from traditional Indonesian fermented foods. Two of those strains converted MSG to GABA after 24 h of incubation at 37 °C, and the most efficient was *L. plantarum* FNCC 260, which yielded a GABA concentration of 1226.5 mg/L in the culture media from 100 mM MSG in the cultivation medium. In the present study, two GABA-producing species were microencapsulated in fresh and freeze-dried form in sodium alginate using vibrating technology with different concentrations of MSG (1% and 2%) as a precursor, with the aim of maximizing GABA production after storage for one month at 4 °C. Results after 48 h of re-inoculation of the microcapsules in MRS broth showed the stability of the microcapsules and the persistence of GABA production until the end of the storage period.

The amount of GABA produced over time agreed with the findings by Tanamool et al., who showed that *L. plantarum* L10-11 isolated from fermented fish produced GABA at a 5-fold higher concentration than the control when 1% MSG (*w*/*v*) was added to the medium [9]. Similarly, Venzuela et al. examined the capacity of 123 species and strains isolated from traditional dairy products manufactured from raw milk to synthesize GABA from 1% MSG and found that, although the GABA production was highly variable (from 0 to 2.61 mM) across the species and strains, 24 LAB strains synthesized more than 1 mM (103.12 mg/L) of GABA, and different strains of *Lactobacillus brevis* and *Lactobacillus plantarum* were among the best producers [34]. Similarly, Lee et al. [35] examined the lactic acid bacteria isolated from kimchi and salt-fermented Jot-gal and found that the strain *Lactobacillus brevis* BJ-20 has the maximum GABA-production ability in MRS broth containing 1% MSG. Using *L. brevis* BJ20, a sea tangle solution was fermented for 5 days to create GABA. The GABA concentration increased dramatically during fermentation, but the glutamic acid concentration declined. These data reveal that *L. brevis* BJ20 converted glutamic acid to GABA in the fermented sea tangle solution [35]. Also, Wu et al. isolated thirty-two LAB strains from nine fish and found that the strain *L. brevis* RK03 in MRS supplemented with 1% MSG showed the highest GABA production (1024 mg/L) [36]. Finally, two GABA-producing strains, *Lactobacillus brevis* PML1 and *Lactobacillus brevis* G42, isolated from different sources, were chosen to be microencapsulated in soy protein isolate alginate using the emulsion method. To screen for GABA production, the isolates were separately cultivated in MRS broth supplemented with 1% (*w*/*v*) MSG. According to the recorded chromatograms, the isolates showed a GABA-producing ability of 304 mg/L and 2511 mg/L after 30 h at 30 °C, respectively. Due to the good cell protection provided by the soy protein isolate–alginate coating, survival and GABA production improved after microencapsulating the bacteria [37]. Regarding the pH of MRS broth after 48 h of incubation of the microcapsules, the reduction found (ranging from 3.7 to 4.3, while the initial pH was 5.8) is in agreement with a study by Li et al. [38].

Interestingly, our results showed that the microencapsulated cells increased the ability to produce GABA in MRS according to the time of storage, up to 1 month. This behavior could be related to some form of adaptation of the cells to the presence of the GABA precursor (MSG). In fact, it is well known that even though the bacterial cells are in a resting condition (4 °C in Ringer in this study), gene expression is not totally dormant. On the other hand, some researchers have acknowledged that too much MSG can inhibit cell growth and reduce GABA production [31].

At 121 °C and pH 8.0, GABA degradation increased by 88% with an increase in treatment time from 15 to 20 min, making its direct incorporation into food formulations difficult [39]. An innovative approach in the functional foods domain is to co-encapsulate probiotics with GABA in order to enhance their bioactive effects [20]. Previous studies have found that microencapsulated probiotic bacteria survive better than non-encapsulated cells during storage [10]. The ideal size of the microcapsules has not been determined because it depends on their use [40]. According to Fareez et al. [41], larger microcapsules provide greater protection against hostile environments. The results of this study proved that the prepared capsules (120 μm) offered superior cell-loading capacity and protection of cells during the storage conditions, as verified by the GABA production during storage. In contrast, Martin et al. concluded that, to prevent a bad sensory impact on the product, the suitable capsule size for food applications should not exceed approximately 80 μm [42].

As the viability of probiotic microorganisms in the final product until consumption is important, the viability of the cells in the microcapsules was determined during storage. A slight increase in plate counts was observed in the case of microcapsules loaded with the freeze-dried mixture, while a slight decrease in viable counts was found with microcapsules prepared with the fresh cell mixture. Similarly, Mahmoud et al. [43] investigated the effect of storage under refrigeration conditions on the viability of *L. plantarum* entrapped in capsules. They found that viable counts increased with all the tested encapsulating agents for 4 weeks. In the present study, chocolate milk has been chosen as an alternative delivery system for microencapsulated GABA-producing bacteria supplemented with MSG to increase stability and productivity. Results showed that the quantity of GABA produced in chocolate milk with microcapsules containing freeze-dried cells was higher than that with fresh cells. These results agreed with the study by Luca and Oroian, who indicated that alginate microcapsules loaded with *L. plantarum* NCIM 2083 exhibited higher survival rates in all the food matrices examined after storage [44]. Moreover, Misra et al. [22] developed symbiotic co-microcapsules containing the probiotic strain *Lactococcus lactis* SKL 13 and a bioactive compound, namely GABA, using spraying and freeze-drying in maltodextrin, dextran, and inulin matrices. The freeze-drying resulted in higher probiotic and GABA encapsulation efficiencies of 95.08 and 90.04%, respectively, than spray-drying (probiotics: 93.12% and GABA: 83.46%). After 60 days of storage, dried capsules showed a 1% reduction in probiotic count and a 5% decrease in GABA content, whereas both powders demonstrated a non-significant (*p* > 0.05) reduction in probiotic counts (2.9 and 1.35 log CFU/mL in intestinal and gastric conditions, respectively) as well as an 80% GABA release after 240 min of simulated gastrointestinal conditions. Due to the reduction in possible chemical reactions that are detrimental to microorganisms, as well as the enhancement of GABA stability in microcapsules, lower storage temperatures enhance the rate of viability [21].

## 5. Conclusions

We can conclude that microencapsulation with MSG and 1 month of storage does not affect the ability of probiotic cells to produce GABA after re-culturing in MRS for 24 h at 37 °C. Moreover, the yield increased in terms of amount of GABA per CFU over the time of storage of up to 1 month. More importantly, after the storage time, the encapsulated cells were able to produce a significant quantity of GABA in chocolate milk over 1 week of storage at 4 °C with negligible pH changes. This ability significantly increased when the cells were encapsulated in freeze-dried from. These results encourage the application of microencapsulation as a method for preserving probiotic cells during storage and for the production of functional foods.

## Figures and Tables

**Table 1:** Viable counts of microencapsulated fresh cells at different times of storage in Ringer at 4 °C.

Strain	MSG %	Before Encapsulation	Time (Weeks)				* p *
			0	1	2	4	
*L. plantarum* LM2	1	1.6 × 10^8^	1.0 × 10^8^	2.6 × 10^7^	7.8 × 10^7^	7.0 × 10^7^	*
	2	1.6 × 10^8^	6.0 × 10^8^	2.9 × 10^7^	9.2 × 10^7^	4.0 × 10^7^	**
*L. brevis* Y1	1	1.3 × 10^8^	8.0 × 10^8^	9.6 × 10^7^	1.6 × 10^7^	4.0 × 10^7^	**
	2	1.3 × 10^8^	1.1 × 10^9^	9.5 × 10^7^	1.2 × 10^7^	2.0 × 10^7^	*

Values are the average of three replicates. Asterisks in the last column indicate a significant difference at *p* < 0.05 (*) or *p* < 0.01 (**), according to the ANOVA test.

**Table 2:** Viable counts of microencapsulated freeze-dried cells at different times of storage in Ringer at 4 °C.

Strain	MSG %	Before Encapsulation	Time (Weeks)				* p *
			0	1	2	4	
*L. plantarum* LM2	1	1.4 × 10^8^	3.0 × 10^7^	1.5 × 10^7^	1.2 × 10^7^	8.5 × 10^7^	**
	2	1.4 × 10^8^	2.0 × 10^7^	9.7 × 10^7^	6.5 × 10^7^	3.8 × 10^7^	*
*L. brevis* Y1	1	2.5 × 10^8^	3.6 × 10^8^	1.1 × 10^7^	1.4 × 10^7^	1.7 × 10^7^	**
	2	2.5 × 10^8^	1.5 × 10^8^	1.3 × 10^7^	1.0 × 10^7^	8.1 × 10^7^	**

Values are the average of three replicates. Asterisks in the last column indicate a significant difference at *p* < 0.05 (*) or *p* < 0.01 (**), according to the ANOVA test.

**Table 3:** GABA production (µg/mL) by fresh and freeze-dried microencapsulated cells after different times of storage in Ringer at 4 °C and further cultivation in MRS broth at 37 °C for 48 h.

Microcapsules	MSG %	Time (Weeks)				*p*
		0	1	2	4	
Fresh cells	1	263.0 ± 2.8	196.5 ± 2.1	155.5 ± 0.7	273.5 ± 0.7	**
	2	250.5 ± 2.1	216 ± 1.4	165.5 ± 0.7	189.5 ± 2.1	**
Freeze-dried cells	1	249.0 ± 1.4	298.5 ± 3.5	311.5 ± 2.1	297.3 ± 1.8	*
	2	224 ± 2.8	308.3 ± 1.8	304.3 ± 1.1	262.0 ± 0.2	**

Values are the average (±SD) of three replicates. Asterisks in the last column indicate a significant difference at *p* < 0.05 (*) or *p* < 0.01 (**), according to the ANOVA test.

**Table 4:** GABA production (µg/mL) from fresh and freeze-dried microencapsulated cells after 1 week in chocolate milk at 4 °C.

Microcapsules	MSG %	
	1	2
Fresh cells	128.3 ± 0.4 ^Aa^	84.8 ± 3.9 ^Ab^
Freeze-dried cells	204.5 ± 5.7 ^Ba^	203.8 ± 1.1 ^Ba^

Values represent the mean (±SD) of three replicates. Different letters within the same column (uppercase) or same row (lowercase) indicate a significant difference at *p* < 0.01, according to the *t*-test.

**Table 5:** Viable count (CFU/mL) of fresh and freeze-dried cells in chocolate milk after one week at 4 °C.

Strain	MSG %	Fresh Cells	Freeze-Dried Cells
*L. plantarum* LM2	1	5.8 × 10^8^	3.8 × 10^8^
	2	8.4 × 10^8^	3.4 × 10^8^
*L. brevis* Y1	1	3.7 × 10^8^	3.2 × 10^8^
	2	2.5 × 10^8^	3.6 × 10^8^

Values are the average of three replicates. No significant difference was found (*p* > 0.05).

## Data Availability

Not applicable.
